# Risk factors for morphine-associated sedation in intravenous patient-controlled analgesia for postoperative pain

**DOI:** 10.1186/s12871-025-03520-1

**Published:** 2025-11-24

**Authors:** Jia-Rong Wu, Hsiang-Ling Wu, Yu-Ming Wu, Juan P. Cata, Jui-Tai Chen, Yih-Giun Cherng, Ying-Hsuan Tai

**Affiliations:** 1 Department of Anesthesiology, Shuang Ho Hospital, Taipei Medical University, 291, Zhongzheng Road, New Taipei City, Zhonghe District 23561 Taiwan; 2 Department of Anesthesiology, School of Medicine, College of Medicine, Taipei Medical University, Taipei, 11031 Taiwan; 3 Department of Anesthesiology, Taipei Veterans General Hospital, Taipei, 11217 Taiwan; 4 School of Medicine, National Yang Ming Chiao Tung University, Taipei, 11221 Taiwan; 5 Department of Anesthesiology and Perioperative Medicine, The University of Texas MD Anderson Cancer Center, 1515 Holcombe Blvd, Unit 409, Houston, TX 77030 USA

**Keywords:** Adverse drug event, Benzodiazepine, Pain control, Opioids, Oversedation

## Abstract

**Background:**

Opioid-related oversedation is associated with respiratory depression and can lead to potentially catastrophic adverse events in hospitalized patients. This study aimed to investigate influential factors for postoperative sedation during morphine-based intravenous patient-controlled analgesia (IV-PCA).

**Methods:**

We enrolled patients who received morphine-based IV-PCA for postoperative pain at a medical hospital between January 2020 and November 2022. The primary outcome was unintentional sedation within 72 h after surgery. The Observer Assessment of Alertness/Sedation Scale (OAA/S) was used to assess the sedation depth at 12-hour intervals. Multivariable logistic regression models were used to calculate the adjusted odds ratio (aOR) with 95% confidence interval (CI) for the outcome of interest.

**Results:**

A total of 1,461 patients were included for analyses. The multivariable analysis identified six independent factors for morphine-associated sedation, including age (aOR: 1.019, 95% CI: 1.008–1.030), current alcohol drinking (aOR: 2.14, 95% CI: 1.24–3.68), preoperative hemoglobin level (aOR: 0.41, 95% CI: 0.21–0.81, on base-2 logarithmic scale), intraoperative use of midazolam (aOR: 1.62, 95% CI: 1.07–2.45), basal morphine infusion (aOR: 2.08, 95% CI: 1.16–3.71), and duration of IV-PCA (aOR: 1.97, 95% CI: 1.09–3.57, on base-2 logarithmic scale). Basal morphine infusion was also associated with moderate-to-deep sedation (OAA/S score ≤ 3) (aOR: 2.75, 95% CI: 1.38–5.46). Furthermore, the combination of intraoperative midazolam use and basal morphine infusion was associated with an increased risk of sedation, suggesting a potential synergistic relationship.

**Conclusion:**

The identified risk factors for morphine-associated oversedation can facilitate early identification and prevention of complications in high-risk populations. Our findings suggest that perioperative use of sedative-hypnotics and continuous opioid infusions should be approached cautiously in at-risk patients.

**Supplementary Information:**

The online version contains supplementary material available at 10.1186/s12871-025-03520-1.

## Introduction

Intravenous patient-controlled analgesia (IV-PCA) is an effective modality for postoperative pain and is widely used in the contemporary era [1, 2]. In IV-PCA, patients are allowed to adjust the analgesic medication through the concept of negative-feedback loop, and the interindividual variability in pharmacokinetic and pharmacodynamic effects is thereby minimized in each patient [3–5]. Mounting evidence has shown that IV-PCA provides better pain relief for postoperative pain and increases patient satisfaction compared with on-demand analgesic regimens [2, 3, 5].

Although IV-PCA offers proven benefits, its reliance on opioids as the primary analgesic is associated with several adverse drug events. Opioid-related adverse effects include sedation and respiratory depression, gastrointestinal tract immobilization, nausea, vomiting, pruritus, and postoperative delirium [4, 6, 7]. Among all adverse drug events, sedation and respiratory distress were the most commonly reported, especially at the start and generally during the first 24 h of opioid therapy [8]. Oversedation could lead to respiratory depression and hypoxia, which may prolong the length of hospital stay and pose a potentially life-threatening risk [9–11].

Opioid-related sedation indicates that patients are at high risk for respiratory depression and related complications, prompting a timely evaluation and intervention [6, 8]. However, the epidemiology of opioid-associated oversedation in IV-PCA remains incompletely understood, with the incidence and risk factors yet to be fully elucidated [4–8]. Therefore, we conducted a retrospective cohort study to evaluate the risk factors associated with unintentional sedation in morphine-based IV-PCA for postoperative pain. This study also aimed to assess the incidence and severity of sedation to better understand the epidemiology of morphine-related sedation in IV-PCA.

## Materials and methods

### Patient evaluation and selection

We obtained the approval of research from the Taipei Medical University-Joint Institutional Review Board, Taipei, Taiwan (TMU-JIRB No. N202205095; date of approval: 9 June 2022). The Institutional Review Board waived the requirement of informed consent due to the use of decoded patient identifications and retrospective nature of this study. This research fully conforms to the ethical standards of the Helsinki Declaration and STROBE Reporting Guidelines [12]. A data analysis and statistical plan was developed and included in the institutional review board protocol prior to accessing the data.

The electronic medical record system was utilized to consecutively include 1,536 patients who underwent surgeries requiring general and/or regional anesthesia, received morphine-based IV-PCA for postoperative pain, and had a postoperative hospital stay of at least 2 days at Shuang Ho Hospital, Taipei Medical University, between January 1, 2020, and November 30, 2022. The exclusion criteria included duplicate cases, missing data on IV-PCA parameters, patients younger than 20 years, switching from patient-controlled epidural anesthesia to IV-PCA, fewer than four postoperative visits for sedation depth assessment, and planned sedation for mechanical ventilation or medical procedures within 72 h after surgery. Data extraction was performed by independent anesthesiologists using a standardized protocol, with these individuals excluded from statistical analyses. Data quality was verified through random sampling by other study authors.

### Anesthesia and analgesia management

Prior to surgery, patients were prohibited from using sedative-hypnotics, except for those who had been prescribed such medications for at least 30 days. For the induction of general anesthesia, fentanyl 1–2 µg·kg^− 1^ and propofol 1–2 mg·kg^− 1^ were used. Neuromuscular blockade for endotracheal intubation was typically achieved using rocuronium 0.6–1.0 mg·kg^− 1^ or cisatracurium 0.1–0.2 mg·kg^− 1^. General anesthesia was maintained using sevoflurane 2–3 vol% or desflurane 6–8 vol%. Neuromuscular blockade was reversed using sugammadex 2 mg·kg^− 1^ or neostigmine 0.05 mg·kg^− 1^. For spinal anesthesia, bupivacaine 6–15 mg without opioids was administered. In combined neuraxial-general anesthesia, a continuous epidural infusion of ropivacaine 1.25–5 mg·ml^− 1^ with or without fentanyl 2.5–5 µg·ml^− 1^ were used. During the intraoperative period, intravenous midazolam 2–5 mg was administered for anxiolysis during the induction of general or spinal anesthesia, tailored to individual patient needs.

Following surgery, IV-PCA was initiated in the post-anesthesia care unit. Patients were contraindicated for IV-PCA if they had the following conditions: conscious disturbance, inadequate cognitive function, and the need for postoperative ventilator support lasting more than 24 h. A portable infusion pump (CADD^®^-Solis Infusion System, Smiths Medical, Inc., Minneapolis, MN, USA) was programmed to deliver morphine sulfate 1 mg·ml^− 1^ in normal saline [13, 14]. IV-PCA pumps were set to deliver a loading dose between 0 and 5.0 ml, a demand dose between 0.5 and 2.0 ml, a basal infusion rate between 0 and 1.5 ml·hr^− 1^, and a lockout time between 5 and 10 min. The pain management team evaluated pain intensity and adverse drug events in patients receiving IV-PCA at 12-hour intervals. IV-PCA was typically administered for 48–72 h before transitioning to oral non-opioid analgesics, such as acetaminophen and nonsteroidal anti-inflammatory drugs.

### Evaluation of sedation depth, morphine dosage, and pain intensity

We employed the Observer Assessment of Alertness/Sedation Scale (OAA/S) to assess sedation depth in patients receiving IV-PCA [15, 16]. The OAA/S is a validated and reliable five-point scale for evaluating sedation depth in adults, defined as follows: 5 (fully awake): responds promptly to name in normal tone, with normal speech and appearance; 4 (mild sedation): lethargic response to name, slight speech slowing, mild ptosis; 3 (moderate sedation): responds only to loud or repeated name calling, with slurred or slowed speech and glazed eyes; 2 (deep sedation): responds only to mild prodding or shaking, with minimal verbal response; 1 (unarousable): no response to mild prodding or shaking [15, 16]. The primary outcome was any sedation within 72 h after surgery, defined as the minimum OAA/S score ≤ 4 during the post-anesthesia visits. The secondary outcome was moderate-to-deep sedation within 72 h after surgery, defined as the minimum OAA/S score of 1 to 3. Given the reported higher sedation incidence associated with basal infusions of IV-PCA [17], we compared sedation depth, morphine consumption, and pain intensity within 72 h after surgery between patients with and without basal morphine infusions. To account for heterogeneity in anesthesia types within this cohort, we analyzed sedation incidence and scores, average pain intensity, and cumulative morphine consumption via IV-PCA across neuraxial, general, and combined anesthesia groups. Certified nurse anesthetists from the pain management team assessed sedation depth, pain intensity, and morphine dosage every 12 h. For patients with an OAA/S score ≤ 3, the level of sedation was re-evaluated and confirmed by the attending anesthesiologist. Postoperative pain intensity was assessed both at rest and during movement and quantified using a self-reported 11-point numeric rating scale (NRS), ranging from “no pain” (0) to “the worst pain imaginable” (10).

### Data collection

To minimize potential confounding effects, the following patient and clinical covariates were collected based on data availability, physiological plausibility, and prior literature [8–11]. Demographic data were age, sex, and body mass index. Preoperative clinical factors were the American Society of Anesthesiologists physical status, current smoking and alcohol drinking (within 30 days before surgery), preoperative use of sedative-hypnotics (within 30 days before surgery), comorbidities (hypertension, diabetes mellitus, major depression, and cancer), and preoperative laboratory testing (hemoglobin, creatinine, aspartate aminotransferase, alanine aminotransferase, and estimated glomerular filtration rate based on the Cockcroft-Gault formula) [18]. Intraoperative covariates were sites of surgery (classified into extremity, upper abdomen, lower abdomen, chest, spine, and other), uses of laparoscopic or robotic techniques, types and duration of anesthesia, volume of blood loss, intravenous fluid volume, intraoperative uses of midazolam, intraoperative opioid consumption, and administration of non-steroidal anti-inflammatory drugs. Postoperative covariates included initial IV-PCA infusion settings (loading dose, demand dose, basal dose, lockout interval, four-hour dose limit, and droperidol use), duration of IV-PCA use, cumulative morphine dose via IV-PCA, postoperative opioid consumption, use of nonsteroidal anti-inflammatory drugs, sedative-hypnotic administration (benzodiazepines and non-benzodiazepines), and regional analgesia application. Morphine milligram equivalents were used to convert the dosages of non-morphine opioids (see Supplementary table S1) [19, 20].

### Sample size estimation

The sample size was determined based on Austin et al., [21]. recommending a minimum of 20 events per variable for logistic regression. With an anticipated sedation incidence of 10% [4–8], 1,461 patients were expected to yield approximately 146 events, sufficient for a multivariable model with up to 7 variables. Our final model included 6 variables, meeting this criterion and ensuring adequate power for detecting clinically meaningful associations.

### Statistical analysis

Kolmogorov–Smirnov and Shapiro–Wilk tests were used to check the normality of the collected data. Logarithmic transformation was used to decrease the distribution skewness of non-normal data, including preoperative laboratory testing, intravenous blood loss and fluid volume, duration of anesthesia, pump infusion parameters, duration, and cumulative morphine dose of IV-PCA, and intraoperative and postoperative opioid consumption. Comparisons of patient characteristics between the sedated and non-sedated patients were performed using chi-square tests or Fisher’s exact tests for categorical variables and either independent t tests or Mann-Whitney U tests for continuous variables, as appropriate. As part of the a priori analysis protocol, logistic regression analyses were conducted to evaluate the association between included variables and morphine-associated sedation. Variables significant in univariate models were adjusted in multivariable models to estimate adjusted odds ratios (aORs) and 95% confidence intervals (CIs) for the outcomes of interest. Collinearity among covariates in the final multivariable models was assessed using variance inflation factors (VIF), with all VIF values below 5, indicating no significant collinearity. Secondary analyses examined sedation incidence, pain scores, and morphine consumption stratified by anesthesia type to explore heterogeneity. Additionally, a sensitivity analysis was performed to compare sedation incidence, morphine consumption, and postoperative NRS pain scores between patients receiving and not receiving basal morphine infusion, utilizing multivariable linear regression models. We used backward stepwise regression analyses with an entry criterion of a significance level of 0.05 and an exit criterion of 0.10 to determine independent explanatory factors for total morphine consumption and pain intensity. Clinical significance for pain score differences was evaluated post-hoc, with a threshold of ≥ 1 point on the NRS considered meaningful, based on prior literature [22]. A two-sided significance level of 0.05 was used to define a statistically significant difference. Statistical analyses were conducted using SAS software, version 9.4 (SAS Institute Inc., Cary, NC, USA). Data graphs were generated using Excel 2019 (Microsoft Corporation, Seattle, WA, USA).

## Results

### Baseline patient characteristics

After meeting the selection criteria, a total of 1,461 patients were included for analysis (Supplementary figure S1). Table 1 shows the baseline patient characteristics between the sedated and non-sedated patients. Patients experiencing morphine-associated sedation were more likely to be older, male, current alcohol users, and have preoperative use of sedative-hypnotics or hypertension. Regarding the intraoperative factors, the sedated patients had longer duration of anesthesia and were more likely to use midazolam compared with the counterparts. In the postoperative period, the sedated patients were more likely to receive basal morphine infusions via IV-PCA, along with sedative-hypnotics and regional analgesia.

**Table 1:** Baseline characteristics of the included patients

	All patients (*n* = 1,461)	Sedated (*n* = 146)	Non-sedated (*n* = 1,315)	*p*
Age, year	49.9 ± 15.7	54.0 ± 18.0	49.5 ± 15.4	0.0044
Sex, male	287 (19.6%)	38 (26.0%)	249 (18.9%)	0.0407
Body mass index, kg·m^− 2^	26.0 ± 4.9	25.8 ± 5.1	26.0 ± 4.9	0.6426
ASA class				0.3448
I	217 (14.9%)	16 (11.0%)	201 (15.3%)	
II	1,208 (82.7%)	127 (87.0%)	1,081 (82.2%)	
III	36 (2.5%)	3 (2.1%)	33 (2.5%)	
Current cigarette smoking	176 (12.1%)	21 (14.4%)	155 (11.8%)	0.3605
Current alcohol drinking	111 (7.6%)	19 (13.0%)	92 (7.0%)	0.0092
Preoperative use of sedative-hypnotics	337 (23.1%)	47 (32.2%)	290 (22.1%)	0.0059
Hypertension	392 (26.8%)	53 (36.3%)	339 (25.8%)	0.0065
Diabetes mellitus	255 (17.5%)	28 (19.2%)	227 (17.3%)	0.5629
Major depression	22 (1.5%)	4 (2.7%)	18 (1.4%)	0.2672
Malignancy	224 (15.3%)	30 (20.6%)	194 (14.8%)	0.0652
Preoperative laboratory testing				
Hemoglobin, g·dL^− 1^	12.5 (11.1–13.7)	12.4 (10.7–13.6)	12.5 (11.2–13.7)	0.1633
eGFR, mL·min·1.73 m^− 2^	96.2 (79.0–111.1)	95.1 (72.0–110.9)	96.2 (80.0–111.1)	0.5538
Alanine aminotransferase, U·L^− 1^	18 (13–28)	19.0 (14.0–29.0)	18.0 (13.0–27.0)	0.2304
Aspartate aminotransferase, U·L^− 1^	20 (16–26)	21.0 (17.0–26.0)	20.0 (16.0–27.0)	0.4232
Surgical site				0.0876
Extremity	301 (20.6%)	41 (28.1%)	260 (19.8%)	
Upper abdomen	140 (9.6%)	15 (10.3%)	125 (9.5%)	
Lower abdomen	841 (57.6%)	71 (48.6%)	770 (58.6%)	
Thorax	22 (1.5%)	1 (0.7%)	21 (1.6%)	
Spine	92 (6.3%)	13 (8.9%)	79 (6.0%)	
Other	65 (4.5%)	5 (3.4%)	60 (4.6%)	
Laparoscopic or robotic surgery	197 (13.5%)	23 (15.8%)	174 (13.2%)	0.3974
Intraoperative blood loss, mL	200 (10–500)	150 (10–400)	200 (10–500)	0.1647
Type of anesthesia				0.7489
Neuraxial anesthesia	471 (32.2%)	43 (29.5%)	428 (32.6%)	
General anesthesia	980 (67.1%)	102 (69.9%)	878 (66.8%)	
Combined general and neuraxial anesthesia	10 (0.7%)	1 (0.7%)	9 (0.7%)	
Anesthesia duration, min	155 (105–235)	185 (110–250)	150 (105–230)	0.0284
Intraoperative fluid volume, mL	900 (600–1,200)	875 (650–1,150)	900 (600–1,200)	0.4190
Intraoperative midazolam	268 (18.3%)	36 (24.7%)	232 (17.6%)	0.0377
Intraoperative opioid consumption, MME	10.0 (3.3–15.0)	10.0 (5.0–15.0)	10.0 (3.3–15.0)	0.5615
Intraoperative use of NSAIDs	206 (14.1%)	26 (17.8%)	180 (13.7%)	0.1748
Initial IV-PCA infusion parameters				
Loading dose, mL	3.0 (2.0–3.0)	3.0 (0–3.0)	3.0 (2.0–3.0)	0.2372
Demand dose, mL	1.1 (1.0–1.5)	1.0 (1.0–1.5)	1.1 (1.0–1.5)	0.6011
Basal dose (binary)	94 (6.4%)	16 (11.0%)	78 (5.9%)	0.0188
Basal dose (continuous), mL·hr^− 1^	0 (0–0)	0 (0–0)	0 (0–0)	0.0201
Lockout interval, min	6 (6–8)	6 (5–8)	6 (6–8)	0.2219
4-hour dose limit, mL	24.0 (20.0–28.0)	20.0 (20.0–28.0)	24.0 (20.0–28.0)	0.1343
Droperidol addition	604 (41.3%)	56 (38.4%)	548 (41.7%)	0.4400
IV-PCA duration, hour	69.1 (63.7–72.4)	70.0 (65.5–73.1)	69.1 (63.5–72.4)	0.0596
IV-PCA cumulative morphine dose, mg	40.3 (22.0–68.9)	42.2 (22.5–72.2)	40.2 (22.0–68.2)	0.2916
Postoperative opioid consumption, MME	40.3 (22.0–69.0)	42.2 (23.0–72.2)	40.2 (22.0–68.4)	0.2834
Postoperative use of NSAIDs	76 (5.2%)	10 (6.9%)	66 (5.0%)	0.3447
Postoperative use of sedative-hypnotics	388 (26.6%)	52 (35.6%)	336 (25.6%)	0.0090
Benzodiazepines	199 (13.6%)	34 (23.3%)	165 (12.6%)	0.0003
Non-benzodiazepine	304 (20.8%)	42 (28.8%)	262 (19.9%)	0.0125
Postoperative regional analgesia	26 (1.8%)	6 (4.1%)	20 (1.5%)	0.0248

Values were mean ± standard deviation, median (interquartile range) or counts (percent)

*ASA* American society of anesthesiologists, *eGFR* Estimated glomerular filtration rate, *IV-PCA* Intravenous patient-controlled analgesia, *MME* Morphine milligram equivalent, *NSAIDs* Non-steroidal anti-inflammatory drugs

### Incidence and depth of sedation

The overall incidence of any and moderate-to-deep sedation was 10.0% and 5.5%, respectively (Table 2). The incidence of any sedation was 2.3%, 3.6%, 3.4%, and 1.6% in the postoperative period of 0–12, 12–36, 36–60, and 60–72 h, respectively. In the study cohort, 90.0%, 4.5%, 4.7%, 0.6%, 0.3% patients had the minimum OAA/S score of 5, 4, 3, 2, and 1 within 72 h after surgery, respectively.

**Table 2:** Incidence and depth of morphine-associated sedation during IV-PCA

	All patients (*n* = 1,461)
Any sedation during POH 0–72	146 (10.0%)
Moderate-to-deep sedation during POH 0–72	81 (5.5%)
POH 0–12	
Any sedation	34 (2.3%)
Moderate-to-deep sedation	18 (1.2%)
OAA/S score = 5	1,427 (97.7%)
OAA/S score = 4	16 (1.1%)
OAA/S score = 3	16 (1.1%)
OAA/S score = 2	1 (0.1%)
OAA/S score = 1	1 (0.1%)
POH 12–36	
Any sedation	52 (3.6%)
Moderate-to-deep sedation	24 (1.6%)
OAA/S score = 5	1,409 (96.4%)
OAA/S score = 4	28 (1.9%)
OAA/S score = 3	21 (1.4%)
OAA/S score = 2	3 (0.2%)
OAA/S score = 1	0 (0)
POH 36–60	
Any sedation	49 (3.4%)
Moderate-to-deep sedation	32 (2.2%)
OAA/S score = 5	1,412 (96.7%)
OAA/S score = 4	17 (1.2%)
OAA/S score = 3	28 (1.9%)
OAA/S score = 2	4 (0.3%)
OAA/S score = 1	0 (0)
POH 60–72	
Any sedation	24 (1.6%)
Moderate-to-deep sedation	13 (0.9%)
OAA/S score = 5	1,437 (98.4%)
OAA/S score = 4	11 (0.8%)
OAA/S score = 3	9 (0.6%)
OAA/S score = 2	1 (0.1%)
OAA/S score = 1	3 (0.2%)
Minimum OAA/S score during POH 0–72	
OAA/S score = 5	1,315 (90.0%)
OAA/S score = 4	65 (4.5%)
OAA/S score = 3	68 (4.7%)
OAA/S score = 2	9 (0.6%)
OAA/S score = 1	4 (0.3%)

Values were counts (percent)

*OAA/S* Observer assessment of alertness/sedation scale, *POH* Postoperative hour

### Risk factors for sedation during IV-PCA

**Fig. 1:**
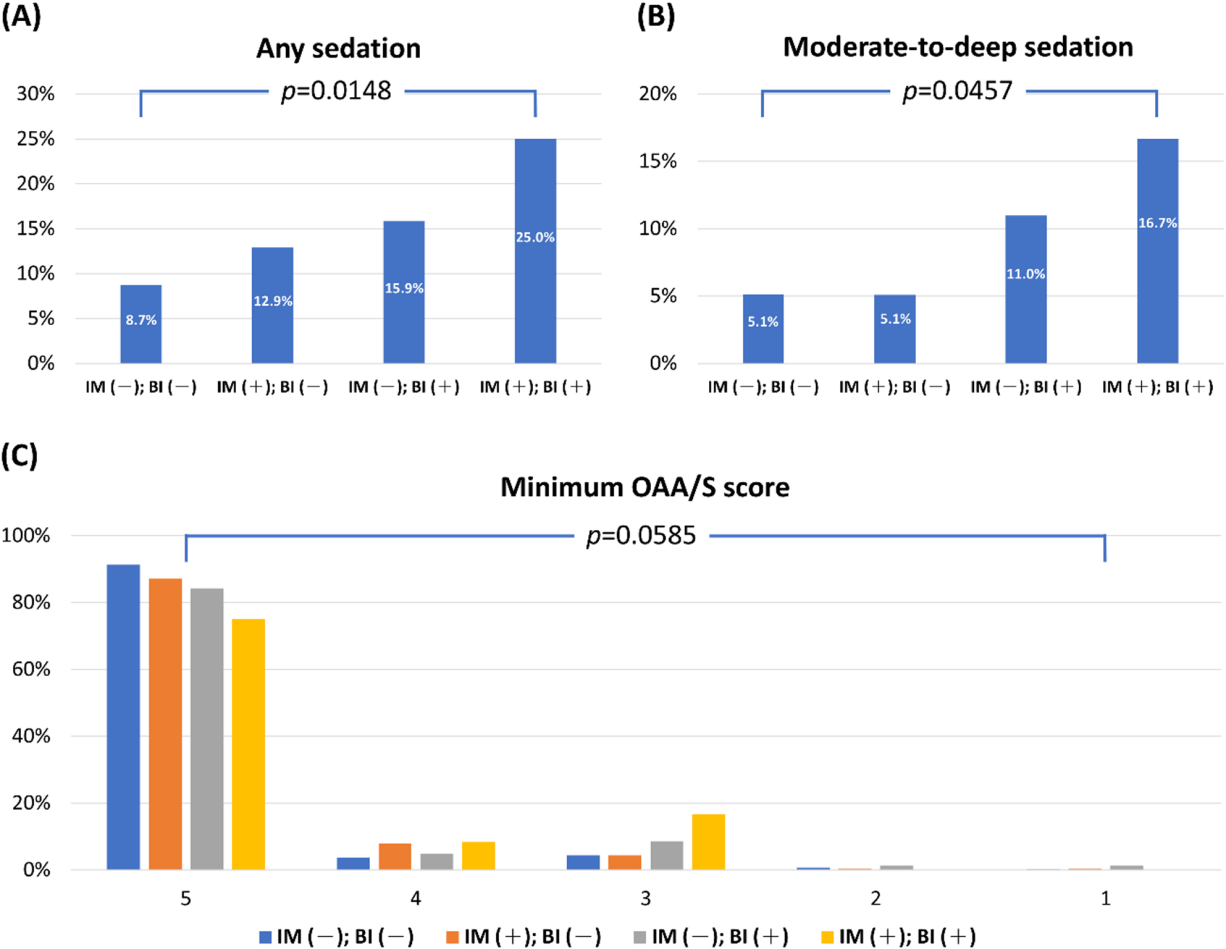
Incidence of any sedation (**A**), moderate-to-deep sedation (**B**), and varying minimum OAA/S scores (**C**) among patients with and without intraoperative use of midazolam or background morphine infusion. BI: background morphine infusion; IM: intraoperative use of midazolam; OAA/S = Observer Assessment of Alertness/Sedation Scale

After adjusting for covariates, multivariable analyses identified six independent factors for any sedation during IV-PCA, including age (aOR: 1.019), current alcohol drinking (aOR: 2.14), preoperative hemoglobin level (aOR: 0.41, on base-2 logarithmic scale), intraoperative use of midazolam (aOR: 1.62), basal morphine infusion (aOR: 2.08), and duration of IV-PCA use (aOR: 1.97, on base-2 logarithmic scale) (Table 3). The univariate analysis determined twelve factors associated with any sedation during IV-PCA, including age (OR: 1.018), sex (male vs. female, OR: 1.51), current alcohol drinking (OR: 1.99), preoperative use of sedative-hypnotics (OR: 1.68), hypertension (OR: 1.64), preoperative hemoglobin level (OR: 0.49, on base-2 logarithmic scale), intraoperative use of midazolam (OR:1.53), basal morphine infusion (OR: 1.95), four-hour dose limit (OR: 0.68, on base-2 logarithmic scale), duration of IV-PCA use (OR: 1.97, on base-2 logarithmic scale), postoperative use of sedative-hypnotics (OR: 1.61), and postoperative regional analgesia (OR: 2.78). Table 4 shows the results of univariate and multivariable analyses for moderate-to-deep sedation. Independent factors for moderate-to-deep sedation were age (aOR: 1.030), preoperative hemoglobin level (aOR: 0.30, on base-2 logarithmic scale), and basal morphine infusion (aOR: 2.75). Fig. 1 illustrates the incidence of any sedation, moderate-to-deep sedation, and varying minimum OAA/S scores among patients with and without intraoperative midazolam use or background morphine infusion. Notably, the combination of intraoperative midazolam use and basal morphine infusion was associated with an increased incidence of any sedation, suggesting a potential synergistic relationship.

**Table 3:** Univariate and multivariable analyses for any sedation during IV-PCA

	Univariate cOR (95% CI)	*p*	Multivariable aOR (95% CI)	*p*
Age, year	1.018 (1.007–1.028)	0.0012	1.019 (1.008–1.030)	0.0005
Sex, male vs. female	1.51 (1.02–2.24)	0.0419	–	–
Body mass index, kg·m^− 2^	0.99 (0.96–1.03)	0.6424	–	–
ASA class		0.3487	–	–
II vs. I	1.48 (0.86–2.54)	0.3444	–	–
III vs. I	1.14 (0.32–4.14)	0.9204	–	–
Current cigarette smoking	1.26 (0.77–2.06)	0.3612	–	–
Current alcohol drinking	1.99 (1.18–3.37)	0.0105	2.14 (1.24–3.68)	0.0062
Preoperative use of sedative-hypnotics	1.68 (1.16–2.43)	0.0063	–	–
Hypertension	1.64 (1.15–2.35)	0.0069	–	–
Diabetes mellitus	1.14 (0.74–1.76)	0.5631	–	–
Major depression	2.03 (0.68–6.08)	0.2060	–	–
Malignancy	1.49 (0.97–2.30)	0.0667	–	–
Preoperative laboratory testing ^†^			–	–
Hemoglobin, g·dL^− 1^	0.49 (0.25–0.95)	0.0348	0.41 (0.21–0.81)	0.0106
eGFR, mL·min·1.73 m^− 2^	0.81 (0.63–1.04)	0.0917	–	–
Alanine aminotransferase, U·L^− 1^	1.08 (0.86–1.35)	0.5057	–	–
Aspartate aminotransferase, U·L^− 1^	1.03 (0.79–1.35)	0.8216	–	–
Surgical site, extremity as reference		0.0951	–	–
Upper abdomen	0.76 (0.41–1.43)	0.5939	–	–
Lower abdomen	0.59 (0.39–0.88)	0.6490	–	–
Thorax	0.30 (0.04–2.31)	0.3745	–	–
Spine	1.04 (0.53–2.05)	0.1332	–	–
Other	0.53 (0.20–1.39)	0.6354	–	–
Laparoscopic or robotic surgery	1.23 (0.76–1.97)	0.3979	–	–
Intraoperative blood loss, mL^†^	0.96 (0.90–1.03)	0.2532	–	–
Type of anesthesia		0.7492	–	–
General vs. neuraxial anesthesia	1.16 (0.80–1.68)	0.8613	–	–
Combined vs. neuraxial anesthesia	1.11 (0.14–8.94)	0.9788	–	–
Anesthesia duration, min^†^	1.24 (0.99–1.55)	0.0568	–	–
Intraoperative fluid volume, mL^†^	0.88 (0.70–1.09)	0.2419	–	–
Intraoperative midazolam	1.53 (1.02–2.28)	0.0389	1.62 (1.07–2.45)	0.0229
Intraoperative opioid consumption, MME^†^	0.89 (0.72–1.10)	0.2797	–	–
Intraoperative use of NSAIDs	1.37 (0.87–2.15)	0.1758	–	–
Initial IV-PCA infusion parameters			–	–
Loading dose, mL^†^	0.92 (0.61–1.36)	0.6612	–	–
Demand dose, mL^†^	0.80 (0.49–1.30)	0.3597	–	–
Basal dose (binary)	1.95 (1.11–3.44)	0.0209	2.08 (1.16–3.71)	0.0139
Basal dose (continuous), mL·hr^− 1†^	0.89 (0.28–2.88)	0.8478	–	–
Lockout interval, min^†^	0.78 (0.46–1.32)	0.3509	–	–
4-hour dose limit, mL^†^	0.68 (0.47–0.98)	0.0363	–	–
Droperidol addition	0.87 (0.61–1.24)	0.4403	–	–
IV-PCA duration, hour^†^	1.97 (1.11–3.49)	0.0205	1.97 (1.09–3.57)	0.0246
IV-PCA cumulative morphine dose, mg^†^	1.08 (0.94–1.24)	0.2961	–	–
Postoperative opioid consumption, MME^†^	1.08 (0.94–1.24)	0.2896	–	–
Postoperative use of NSAIDs	1.39 (0.70–2.77)	0.3458	–	–
Postoperative use of sedative-hypnotics	1.61 (1.12–2.31)	0.0095	–	–
Postoperative regional analgesia	2.78 (1.10–7.03)	0.0312	–	–

*aOR* Adjusted odds ratio, *ASA* American society of anesthesiologists, *CI* Confidence interval, *cOR* Crude odds ratio, *eGFR* Estimated glomerular filtration rate, *IV-PCA* Intravenous patient-controlled analgesia, *MME* Morphine milligram equivalent, *NSAIDs* Non-steroidal anti-inflammatory drugs

†On base-2 logarithmic scale

**Table 4:** Univariate and multivariable analyses for moderate-to-deep sedation during IV-PCA

	Univariate cOR (95% CI)	*p*	Multivariable aOR (95% CI)	*p*
Age, year	1.029 (1.015–1.043)	< 0.0001	1.030 (1.016–1.044)	< 0.0001
Sex, male vs. female	1.79 (1.09–2.94)	0.0215	–	–
Body mass index, kg·m^− 2^	1.02 (0.97–1.06)	0.4362	–	–
ASA class		0.2804	–	–
II vs. I	1.90 (0.86–4.19)	0.4032	–	–
III vs. I	1.77 (0.35–8.85)	0.7439	–	–
Current cigarette smoking	1.03 (0.52–2.04)	0.9315	–	–
Current alcohol drinking	1.57 (0.76–3.22)	0.2231	–	–
Preoperative use of sedative-hypnotics	1.94 (1.21–3.11)	0.0059	–	–
Hypertension	1.96 (1.24–3.10)	0.0042	–	–
Diabetes mellitus	1.38 (0.80–2.37)	0.2458	–	–
Major depression	1.72 (0.40–7.50)	0.4692	–	–
Malignancy	2.03 (1.21–3.41)	0.0075	–	–
Preoperative laboratory testing ^†^			–	–
Hemoglobin, g·dL^− 1^	0.35 (0.15–0.81)	0.0146	0.30 (0.13–0.71)	0.0060
eGFR, mL·min·1.73 m^− 2^	0.64 (0.49–0.83)	0.0007	–	–
Alanine aminotransferase, U·L^− 1^	1.12 (0.86–1.47)	0.3950	–	–
Aspartate aminotransferase, U·L^− 1^	1.27 (0.93–1.75)	0.1366	–	–
Surgical site, extremity as reference		0.0625	–	–
Upper abdomen	1.56 (0.76–3.19)	0.0899	–	–
Lower abdomen	0.61 (0.35–1.07)	0.1193	–	–
Thorax	0.67 (0.09–5.23)	0.7187	–	–
Spine	1.34 (0.57–3.15)	0.3044	–	–
Other	0.68 (0.20–2.36)	0.5772	–	–
Laparoscopic or robotic surgery	1.50 (0.84–2.68)	0.1749	–	–
Intraoperative blood loss, mL^†^	0.96 (0.88–1.05)	0.3610	–	–
Type of anesthesia		0.4364	–	–
General vs. neuraxial anesthesia	1.40 (0.84–2.33)	0.9725	–	–
Combined vs. neuraxial anesthesia	< 0.01 (< 0.01 – >999.99)	0.9737	–	–
Anesthesia duration, min^†^	1.33 (1.00–1.78)	0.0514	–	–
Intraoperative fluid volume, mL^†^	1.19 (0.99–1.43)	0.0720	–	–
Intraoperative midazolam	1.01 (0.57–1.80)	0.9665	–	–
Intraoperative opioid consumption, MME^†^	0.98 (0.74–1.30)	0.9076	–	–
Intraoperative use of NSAIDs	1.39 (0.78–2.49)	0.2634	–	–
Initial IV-PCA infusion parameters			–	–
Loading dose, mL^†^	0.87 (0.51–1.47)	0.5903	–	–
Demand dose, mL^†^	0.60 (0.32–1.15)	0.1254	–	–
Basal dose (binary)	2.46 (1.25–4.82)	0.0089	2.75 (1.38–5.46)	0.0039
Basal dose (continuous), mL·hr^− 1†^	1.04 (0.24–4.43)	0.9584	–	–
Lockout interval, min^†^	0.83 (0.42–1.65)	0.5950	–	–
4-hour dose limit, mL^†^	0.70 (0.44–1.14)	0.1527	–	–
Droperidol addition	0.92 (0.58–1.46)	0.7301	–	–
IV-PCA duration, hour^†^	2.10 (0.96–4.59)	0.0631	–	–
IV-PCA cumulative morphine dose, mg^†^	1.19 (0.99–1.43)	0.0720	–	–
Postoperative opioid consumption, MME^†^	1.19 (0.99–1.43)	0.0721	–	–
Postoperative use of NSAIDs	0.93 (0.33–2.61)	0.8919	–	–
Postoperative use of sedative-hypnotics	1.68 (1.05–2.68)	0.0295	–	–
Postoperative regional analgesia	2.27 (0.67–7.72)	0.1893	–	–

*aOR* Adjusted odds ratio, *ASA* American society of anesthesiologists, *CI* Confidence interval, *cOR* Crude odds ratio, *eGFR* Estimated glomerular filtration rate, *IV-PCA* Intravenous patient-controlled analgesia, *MME* Morphine milligram equivalent, *NSAIDs* Non-steroidal anti-inflammatory drugs

†On base-2 logarithmic scale

### Impact of basal morphine infusion on oversedation and pain control

Basal morphine infusions were associated with a higher incidence of any sedation and moderate-to-deep sedation compared with no basal infusion in IV-PCA, 17.0% vs. 9.5% (*p* = 0.0188) and 11.7% vs. 5.1% (*p* = 0.0070), respectively (Table 5). Basal morphine infusions were also linked to lower minimum OAA/S scores (*p* = 0.0448). The cumulative morphine dose of IV-PCA in patients with basal morphine infusion was significantly higher than that of patients without, median 75.7 mg and 38.0 mg (*p* < 0.0001), respectively. Although the mean daily maximum NRS pain scores were slightly lower in patients receiving basal morphine infusions, this difference possibly represents a low clinical significance in daily practice, median 2.8 vs. 2.9 (*p* = 0.0229). Multivariable linear regression analyses revealed that basal morphine infusions were associated with increased cumulative morphine consumption (adjusted *β* = 0.997, *p* < 0.0001) but not with postoperative average NRS pain scores (adjusted *β* = −0.090, *p* = 0.0661) (Supplementary tables S2 and S3).

**Table 5:** Incidence and depth of sedation, postoperative cumulative morphine dose, and average pain scores among patients with or without receiving a basal morphine infusion

	Basal Infusion (*n* = 94)	No Basal Infusion (*n* = 1,367)	*p*
Any sedation during POH 0–72	16 (17.0%)	130 (9.5%)	0.0188
Moderate-to-deep sedation during POH 0–72	11 (11.7%)	70 (5.1%)	0.0070
Minimum OAA/S score during POH 0–72			0.0448
OAA/S score = 5	78 (83.0%)	1,237 (90.5%)	
OAA/S score = 4	5 (5.3%)	60 (4.4%)	
OAA/S score = 3	9 (9.6%)	59 (4.3%)	
OAA/S score = 2	1 (1.1%)	8 (0.6%)	
OAA/S score = 1	1 (1.1%)	3 (0.2%)	
Cumulative morphine dose of IV-PCA, mg	75.7 (45.3, 94.5)	38.0 (21.0, 65.4)	< 0.0001
	79.2 ± 44.2	49.8 ± 45.3	
Mean daily maximum NRS pain score	2.8 (2.1, 3.3)	2.9 (2.4, 3.5)	0.0229
	2.7 ± 0.8	3.0 ± 0.9	
Daily maximum NRS pain score			
POH 0–12, at rest	3.0 (2.0, 4.0)	3.0 (2.0, 4.0)	0.6804
	2.9 ± 1.6	2.9 ± 1.6	
POH 0–12, during movement	4.0 (3.0, 6.0)	5.0 (4.0, 6.0)	0.3698
	4.4 ± 1.7	4.5 ± 2.0	
POH 12–36, at rest	2.0 (2.0, 3.0)	2.0 (1.0, 3.0)	0.7342
	2.3 ± 1.2	2.4 ± 1.4	
POH 12–36, during movement	4.0 (3.0, 5.0)	4.0 (3.0, 6.0)	0.3755
	4.3 ± 1.6	4.5 ± 1.8	
POH 36–60, at rest	1.0 (1.0, 2.0)	2.0 (1.0, 2.0)	0.0546
	1.5 ± 1.0	1.8 ± 2.0	
POH 36–60, during movement	3.0 (2.0, 4.0)	3.0 (3.0, 5.0)	0.0135
	3.3 ± 1.3	3.7 ± 1.6	
POH 60–72, at rest	1.0 (0.0, 1.0)	1.0 (0.0, 2.0)	0.0222
	0.9 ± 0.9	1.1 ± 1.0	
POH 60–72, during movement	2.0 (1.0, 3.0)	2.0 (2.0, 3.0)	0.0099
	2.2 ± 1.5	2.6 ± 1.5	

Values are median (interquartile range) or mean ± standard deviation. Continuous variables were compared between groups using Wilcoxon rank-sum tests

*IV-PCA* Intravenous patient-controlled analgesia, *NRS* Numeric rating scale, *OAA/S* Observer assessment of alertness/sedation scale, *POH* Postoperative hour

### Sedation depth, morphine consumption, and pain scores in different anesthesia types

Supplementary table S4 presents the postoperative sedation incidence and scores, NRS pain scores, and cumulative morphine consumption, stratified by anesthesia type. Notably, sedation incidence and depth did not differ across neuraxial, general, and combined anesthesia groups. However, patients receiving general anesthesia reported higher average pain scores and greater cumulative morphine consumption compared to those in the other groups.

## Discussion

This retrospective cohort study elucidates key risk factors for unintentional sedation in morphine-based IV-PCA, significantly advancing perioperative pain management. We identified intraoperative midazolam use and basal morphine infusion as modifiable risk factors for sedation. Notably, basal morphine infusion was associated with significantly higher morphine consumption but lacked a meaningful association with postoperative NRS pain scores, indicating limited analgesic benefit. These findings support the cautious use of sedative-hypnotics and basal opioid infusions, particularly in high-risk patients, such as the elderly or those with comorbidities. Few studies have prioritized sedation as the primary outcome in IV-PCA, leaving its epidemiology and risk factors largely underexplored [4–8]. By demonstrating the synergistic effects of midazolam and basal infusion, this study fills a critical gap in the literature, offering actionable insights for risk stratification and safer IV-PCA protocols to prevent oversedation and related complications.

Several studies have reported that continuous IV-PCA infusions may elevate the risk of opioid-related adverse effects, including respiratory depression [7, 23–25]. Consistent with these findings, our data demonstrated that basal IV-PCA infusions were associated with higher total opioid doses and increased sedation incidence, without a significant reduction in postoperative pain intensity. These results suggest that routine basal opioid infusions should be avoided in surgical patients, particularly opioid-naïve individuals and the elderly [26]. However, two meta-analyses found no significant differences in opioid consumption or sedation risk between pediatric patients with and without basal infusions [24, 27]. This discrepancy may stem from variations in patient demographics (e.g., pediatric vs. adult), surgical and anesthesia types, and sedation assessment methods. Additionally, our study observed that prolonged IV-PCA use was linked to a higher risk of morphine-associated sedation, potentially reflecting greater pain relief needs in patients undergoing extensive surgeries or those with heightened pain sensitivity, which may increase postoperative sedation risk.

Benzodiazepines, such as midazolam, are frequently administered intraoperatively to alleviate anxiety and induce amnesia, facilitating surgical procedures [28, 29]. In our cohort, 18.3% of patients received intraoperative midazolam for light sedation or anxiolysis. Evidence suggests that co-administration of benzodiazepines and opioids may heighten the risk of opioid overdose [26, 28, 30–32]. Both drug classes share similar adverse effects, including sedation and respiratory depression, despite distinct mechanisms of action [28, 31]. While benzodiazepines enhance γ-aminobutyric acid (GABA)-mediated inhibition and opioids activate mu-opioid receptors, both contribute to central nervous system depression [28–30]. As benzodiazepines and opioids are metabolized by cytochrome P450 (CYP) enzymes, clinicians should avoid prescribing CYP inhibitors concurrently to minimize the risk of sedation and respiratory depression [33].

Advanced age is associated with an elevated risk of opioid-induced adverse events [34]. Aging leads to physiological declines in renal, hepatic, and nervous system functions [35, 36], alongside reduced total body water and increased body lipid composition [34, 37]. Impaired renal and hepatic functions hinder opioid metabolism and clearance, while altered body composition, including changes in lipid metabolism and plasma volume, affects opioid distribution and serum concentrations. These pharmacokinetic changes prolong opioid effects, heightening the risk of adverse events. A randomized trial reported that 17.5% of elderly patients experienced difficulties initiating patient-controlled analgesia due to altered mental status, such as confusion or delirium, on the day of surgery [38]. Clinicians should therefore consider the potential for cognitive impairment when prescribing patient-controlled analgesia for elderly patients.

Low preoperative hemoglobin levels may indicate malnutrition in surgical patients [39, 40]. Poor nutritional status is associated with reduced serum protein levels, which can decrease protein-bound morphine, thereby increasing its potency [41]. A prospective study by Tian et al. found that severely malnourished patients, identified using the Nutritional Risk Screening Tool 2002, required 16.8% less propofol for anesthesia induction and experienced a 6.8-minute longer recovery time compared to those with normal nutritional status [42]. These effects may stem from altered drug metabolism, distribution, and clearance due to malnutrition [42]. Anemia may also arise from renal or hepatic dysfunction, which are critical for analgesic metabolism and elimination [43, 44]. However, our analysis found no significant association between estimated glomerular filtration rate, alanine aminotransferase, aspartate aminotransferase levels, and sedation risk. Further research is needed to elucidate the interplay among hemoglobin levels, nutritional status, and opioid-related adverse events.

Alcohol, a known central nervous system depressant, enhances the inhibitory effects of GABA-A receptors and suppresses excitatory N-methyl-D-aspartate receptor activity by competing with amino acid binding [31, 45]. However, the interaction between alcohol and opioids, particularly its impact on sedation, remains incompletely understood. A prior review suggests that alcohol amplifies and synergizes with opioid-induced inhibitory signaling, potentially leading to sedation and respiratory depression [46]. Recent evidence implicates Toll-like receptors (TLRs) and myeloid differentiation primary response gene 88 (MyD88)-dependent pathways in non-neuronal central nervous system effects of both alcohol and opioids, indicating a potential drug-drug interaction [46]. An animal study further identified the TLR2–MyD88–NF-κB signaling pathway as a key mediator of acute sedative effects in alcohol-morphine interactions [47]. Our findings suggest that IV-PCA should be administered cautiously in patients with current alcohol consumption, with infusion rates and regimens tailored individually to minimize sedation risk.

Our analyses identified several patient characteristics linked to sedation during morphine-based IV-PCA, including advanced age, current alcohol consumption, and low preoperative hemoglobin levels, which may facilitate risk stratification and early prevention. Two modifiable risk factors were also identified, including intraoperative midazolam use and basal morphine infusion. Notably, the combination of these factors was associated with a heightened risk of sedation, suggesting a synergistic effect. For high-risk patients, more frequent monitoring and individualized dose titration based on clinical response are essential to prevent deep sedation.

Patients with heightened sensitivity to opioid-induced sedation require special attention. Prior studies indicate that advanced age, female sex, obesity, and comorbidities such as sleep apnea, chronic obstructive pulmonary disease, and substance use disorders increase the risk of oversedation and respiratory depression, even at low pain levels and opioid doses [48, 49]. To mitigate these risks, clinicians should carefully titrate opioid doses in vulnerable populations and consider opioid-sparing strategies, such as adjuvant non-opioid analgesics and regional anesthesia [50]. Enhanced monitoring of sedation and respiratory parameters is critical, particularly within the first 24 postoperative hours [8]. Further research is needed to determine optimal IV-PCA regimens and infusion settings that balance analgesic efficacy with safety in these patients.

This retrospective study is subject to several limitations inherent to its design. First, our analysis could only establish associations between risk factors and unintentional sedation in IV-PCA, necessitating prospective randomized trials to confirm causality for modifiable factors. Second, sedation depth was assessed by certified nurse anesthetists at 12-hour intervals using a standardized scale. Despite this, the subjective nature of the assessment may introduce variability among evaluators. Objective electroencephalogram-based monitoring, such as bispectral index or spectral entropy, could reduce measurement bias. Moreover, the 12-hour evaluation intervals may overlook transient oversedation events, potentially underestimating incidence. Third, we did not analyze other analgesic modalities (e.g., acetaminophen, gabapentinoids, ketamine) due to their infrequent use in IV-PCA patients at our institution. Fourth, several previously identified sedation risk factors, including obstructive sleep apnea, frailty, chronic pain, and opioid tolerance, were not examined due to incomplete data [48, 49]. Finally, the absence of routine respiratory monitoring precluded estimation of respiratory depression incidence, leaving its relationship with oversedation and oxygen desaturation unclear.

In conclusion, our study identified key patient characteristics linked to unintentional sedation during morphine-based IV-PCA, including advanced age, current alcohol consumption, and low preoperative hemoglobin levels. These factors can guide risk stratification and inform tailored IV-PCA dosing and regimens. We also identified intraoperative midazolam use and basal morphine infusion as modifiable risk factors for oversedation. Notably, basal infusions were not associated with enhanced analgesia but were linked to higher opioid consumption, potentially increasing the risk of opioid overdose and related adverse events. Consequently, clinicians should minimize perioperative sedative-hypnotic use and avoid continuous opioid infusions in high-risk patients to prevent oversedation and associated complications.

## Supplementary Information

Supplementary Material 1.

## Data Availability

The data from this study are available upon request from the corresponding author. They are not publicly accessible due to Institutional Review Board regulations.
