# Supplementary material for: Risk factors for morphine-associated sedation in intravenous patient-controlled analgesia for postoperative pain
Source: BMC Anesthesiol. 2025 Nov 24;26:2. doi: 10.1186/s12871-025-03520-1 (PMC12764038; doi:10.1186/s12871-025-03520-1)

Supplementary Table S1: Opioid equianalgesic conversion chart

| **Opioid** | **Administration route** | **Dose equivalent to 1 mg of parenteral morphine (mg)** |
| --- | --- | --- |
| Morphine | Oral | 3 |
| Fentanyl | Intravenous | 0.01 |
| Alfentanil | Intravenous | 0.087 |
| Remifentanil | Intravenous | 0.01 |
| Tramadol | Intravenous | 7.5 |
| Tramadol | Oral | 15 |
| Meperidine | Intravenous | 7.5 |

Supplementary Table S2: Multivariable analyses for cumulative morphine consumption of IV-PCA

|  | **Unstandardized β**  **(95% CI)** | **SE** | **Standardized β** | ***p*** |
| --- | --- | --- | --- | --- |
| **Use of IV-PCA basal dose** | 0.997 (0.755, 1.239) | 0.123 | 0.193 | <.0001 |
| **IV-PCA demand dose, mL^†^** | 0.630 (0.452, 0.807) | 0.090 | 0.175 | <.0001 |
| **Age** | -0.011 (-0.016, -0.006) | 0.003 | -0.139 | <.0001 |
| **Sex, male vs. female** | 0.288 (0.109, 0.467) | 0.091 | 0.091 | 0.0016 |
| **Current cigarette smoking** | 0.322 (0.123, 0.522) | 0.102 | 0.083 | 0.0016 |
| **Laparoscopic or robotic surgery** | -0.263 (-0.454, -0.072) | 0.097 | -0.071 | 0.0070 |
| **Intraoperative fluid volume, mL^†^** | 0.141 (0.058, 0.224) | 0.042 | 0.084 | 0.0009 |
| **Postoperative use of sedative-hypnotics** | 0.221 (0.076, 0.366) | 0.074 | 0.077 | 0.0028 |
| **Surgical site** |  |  |  |  |
| Upper abdomen vs. extremity | 0.855 (0.605, 1.106) | 0.128 | 0.200 | <.0001 |
| Lower abdomen vs. extremity | 0.389 (0.194, 0.584) | 0.099 | 0.152 | <.0001 |
| Thorax vs. extremity | -0.016 (-0.514, 0.482) | 0.254 | -0.002 | 0.9502 |
| Spine vs. extremity | 0.546 (0.273, 0.818) | 0.139 | 0.105 | <.0001 |
| Other vs. extremity | -0.365 (-0.677, -0.053) | 0.159 | -0.060 | 0.0220 |
| **Type of anesthesia** |  |  |  |  |
| General vs. neuraxial anesthesia | -0.329 (-0.481, -0.178) | 0.077 | -0.123 | <.0001 |
| Combined vs. neuraxial anesthesia | -0.534 (-1.257, 0.189) | 0.369 | -0.035 | 0.1479 |

β = regression coefficient; SE = standard error of β; Factors with β > 0 increased total cumulative dose of IV-PCA and those with beta < 0 reduced it. Model fit: R^2^ = 0.1979, adjusted R^2^ = 0.1896. CI, confidence interval. † On base-2 logarithmic scale

Supplementary Table S3: Multivariable analyses for postoperative average NRS pain scores

|  | **Unstandardized β**  **(95% CI)** | **SE** | **Standardized β** | ***p*** |
| --- | --- | --- | --- | --- |
| **Use of IV-PCA basal dose** | -0.090 (-0.186, 0.006) | 0.049 | -0.048 | 0.0661 |
| **Current cigarette smoking** | 0.101 (0.027, 0.175) | 0.038 | 0.070 | 0.0075 |
| **Cancer history** | 0.092 (0.022, 0.163) | 0.036 | 0.072 | 0.0106 |
| **Postoperative use of sedative-hypnotics** | 0.112 (0.055, 0.168) | 0.029 | 0.106 | 0.0001 |
| **Surgical site** |  |  |  |  |
| Upper abdomen vs. extremity | 0.180 (0.083, 0.277) | 0.049 | 0.114 | 0.0003 |
| Lower abdomen vs. extremity | 0.015 (-0.049, 0.078) | 0.032 | 0.015 | 0.6549 |
| Thorax vs. extremity | -0.006 (-0.206, 0.193) | 0.102 | -0.002 | 0.9502 |
| Spine vs. extremity | 0.050 (-0.057, 0.157) | 0.055 | 0.026 | 0.3636 |
| Other vs. extremity | -0.026 (-0.154, 0.102) | 0.065 | -0.011 | 0.6939 |

β = regression coefficient; SE = standard error of β; Factors with β > 0 increased total cumulative dose of IV-PCA and those with beta < 0 reduced it. Model fit: R^2^ = 0.0410, adjusted R^2^ = 0.0350. CI = confidence interval.

Supplementary Table S4: Sedation rates and scores, NRS pain scores, and cumulative morphine consumption during the postoperative hours 0 to 72, stratified by types of anesthesia

|  | **NA**  **(*n* = 471)** | **GA**  **(*n* = 980)** | **Combined**  **NA and GA**  **(*n* = 10)** | ***p*** |
| --- | --- | --- | --- | --- |
| **Any sedation** | 43 (9.1%) | 102 (10.4%) | 1 (10.0%) | 0.7489 |
| **Moderate-to-deep sedation** | 21 (4.5%) | 60 (6.1%) | 0 (0) | 0.3210 |
| **Minimum OAA/S score** |  |  |  | 0.2420 |
| OAA/S score = 5 | 428 (90.9%) | 878 (89.6%) | 9 (90.0%) |  |
| OAA/S score = 4 | 22 (4.7%) | 42 (4.3%) | 1 (10.0%) |  |
| OAA/S score = 3 | 15 (3.2%) | 53 (5.4%) | 0 (0) |  |
| OAA/S score = 2 | 3 (0.6%) | 6 (0.6%) | 0 (0) |  |
| OAA/S score = 1 | 3 (0.6%) | 1 (0.1%) | 0 (0) |  |
| **Average NRS pain scores** | 2.8 (2.3 – 3.3) | 3.0 (2.4 – 3.5) | 2.6 (2.0 – 3.0) | <.0001 |
| **IV-PCA cumulative morphine consumption, mg** | 49.0 (28.0 – 77.5) | 36.0 (20.0 – 63.4) | 23.8 (11.9 – 68.0) | <.0001 |

Values were median (interquartile range) or counts (percent). IV-PCA = intravenous patient-controlled analgesia; NE = neuraxial anesthesia; NRS = numeric rating scale; GA = general anesthesia; OAA/S = Observer Assessment of Alertness/Sedation Scale.

Supplementary Figure S1: Flow diagram for patient inclusion


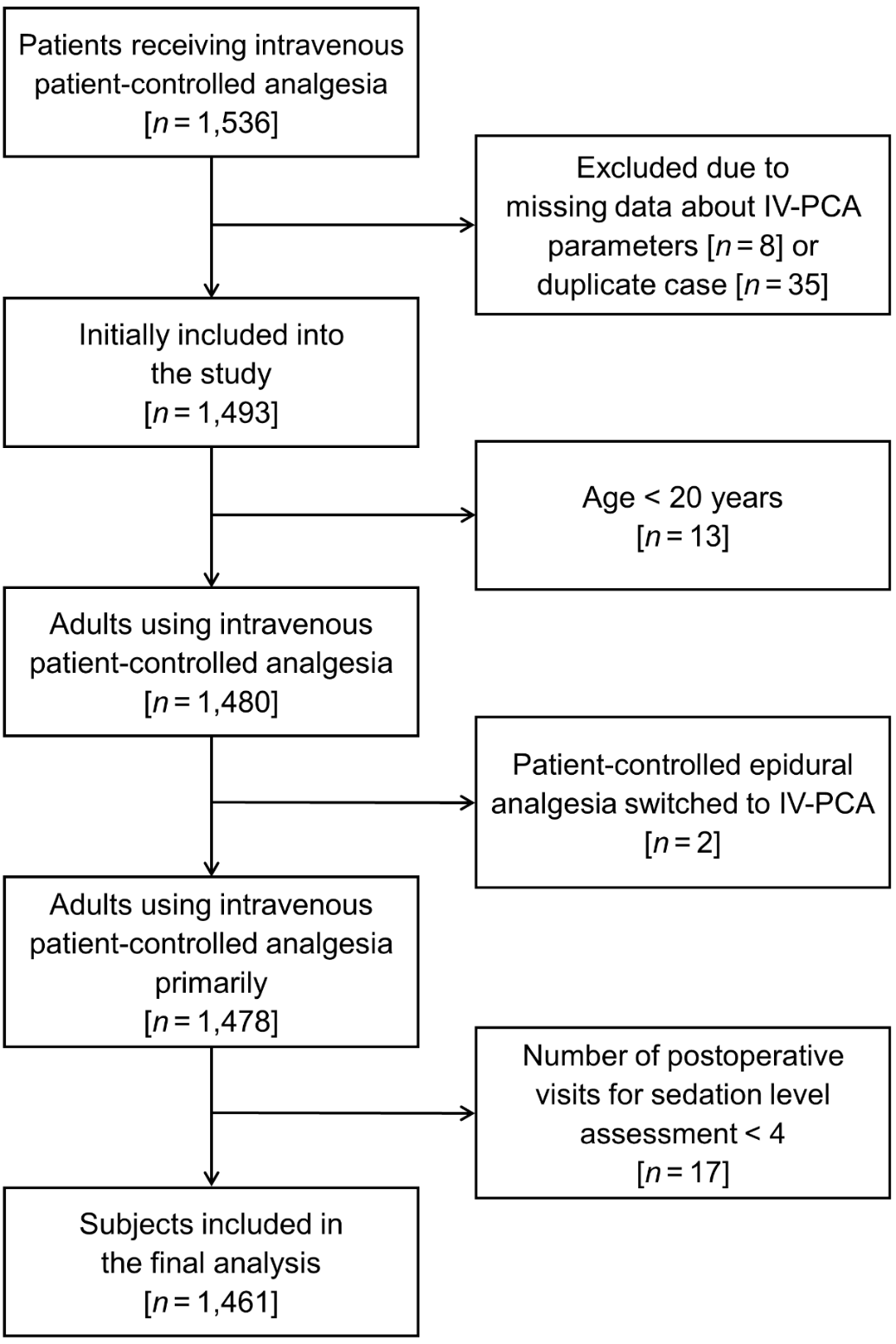

Supplement: Supplementary file 1 — Supplementary Material 1. [file 12871_2025_3520_MOESM1_ESM.docx]
